# A Novel Method for the Quantification of White Wine Mannoproteins by a Competitive Indirect Enzyme-Linked Lectin Sorbent Assay (CI-ELLSA)

**DOI:** 10.3390/molecules23123070

**Published:** 2018-11-23

**Authors:** Matteo Marangon, Mara Vegro, Simone Vincenzi, Giovanna Lomolino, Alberto De Iseppi, Andrea Curioni

**Affiliations:** 1 Department of Agronomy, Food, Natural Resources, Animals and Environment (DAFNAE), University of Padova, Viale dell’Università 16, 35020 Padova, Italy; matteo.marangon@unipd.it (M.M.); mara.vegro@unipd.it (M.V.); giovanna.lomolino@unipd.it (G.L.); alberto.deiseppi@phd.unipd.it (A.D.I.); andrea.curioni@unipd.it (A.C.); 2 Centre for Research in Viticulture and Enology (CIRVE), Viale XXVIII Aprile 14, 31015 Conegliano, Italy

**Keywords:** wine, mannoproteins, competitive indirect enzyme-linked lectin sorbent assay (CI-ELLSA), method, quantification, yeast invertase

## Abstract

Mannoproteins (MPs) are cell wall proteoglycans released in wine by yeast during fermentation and ageing on lees, a procedure used for the production of several wines to enrich them in these components with consequences from both a technological and sensory point of view. Given the significance that wine MPs have for wine quality, winemakers would welcome a simple and accurate method for their quantification, as this would allow them to have a better control of this aspect at different winemaking stages. This study develops and validates a novel, simple and accurate method for MPs quantification in white wines based on a competitive indirect enzyme-linked lectin sorbent assay (CI-ELLSA), using the highly mannosylated yeast invertase as the standard. The method utilizes the lectin concanavalin A (ConA) as the immobilized ligand for MPs, and peroxidase, an enzyme rich in mannose, as the competitor for ConA. After addition of the peroxidase substrate, the intensity of the signal produced by the activity of this enzyme (absorbance at 450 nm) is inversely proportional to the amount of mannosylated proteins in the sample. Results have been validated on several wine styles including still, sparkling and sweet wines.

## 1. Introduction

Mannoproteins (MPs) are the second most abundant class of polysaccharides found in wine [1,2,3]. They are located in the outermost layer of the yeast cell wall and can account for up to 50% of the cell wall dry mass of *Saccharomyces cerevisiae* [4]. Mannoproteins are made of high quantities of mannose (>50%) and exist as covalent complexes with small amounts of protein [4]. Wine MPs generally have protein contents ranging between 1% and 10% [1,5], and have been reported to have sizes that vary within the range 5–800 kDa [6], with typical range between 50 and 500 kDa [1]. Mannoproteins can be released into the wine in two ways as the yeast can either secrete them directly during the alcoholic fermentation, or indirectly during the autolytic phase thanks to the action of yeast β-1,3 glucanases that “disassemble” the cell wall thus releasing these proteoglycans into the wine [7,8,9,10,11]. The presence of MPs in wines has great relevance from both a technological and sensorial point of view [12] as they can have direct or indirect effects upon several wine characteristics. In particular, MPs can: (i) act as protective colloids thus increasing the wine heat [13,14,15] and cold stabilities [16]; (ii) contribute to the growth of malolactic bacteria [17]; (iii) reinforce the aromatic components of a wine [18,19]; (iv) contribute to the gustatory sensations of texture, roundness and mouthfeel [20,21,22]; (v) participate in the formation and stabilization of sparkling wine foams [7,12,23].

Given the relevance of MPs for these aspects, it is of paramount importance to have a simple and accurate method for their quantification in wine. However, today a limited number of methods have been proposed. These methods mostly rely on preliminary MPs separation step(s) generally by firstly isolating the total polysaccharides from the wine (e.g., by ethanol precipitation, concentration by ultrafiltration, chemical extraction [7]), followed by affinity chromatography with the lectin concanavalin A (ConA) as the binding agent [1,5,7,24]. This approach has been widely used as it is the most effective in separating the mannosylated from the non-mannosylated polysaccharidic fractions. Once separated, MPs are quantified by different methods which are rather complicated, time consuming, and generally requiring sophisticated laboratory facilities. Probably the most used approach to this aim relies on the analysis of the sugar composition and quantification by gas chromatography (GC) of the partially methylated alditol acetates. For example, following an approach used by others [25,26], Guadalupe et al. [27] developed a method for the quantification of polysaccharide classes including MPs in musts and wines based on the concentration of total polysaccharides by ethanol precipitation followed by their acidic methanolysis and derivatization using trimethylsilyl (TMS) ester O-methyl glycolsyl derivatives. The analysis of the derivatized monosaccharides carried out by gas chromatography-mass spectrometry (GC-MS) and gas chromatography with flame ionization detector (GC-FID) showed that different classes of polysaccharides could be accurately quantified, with MPs content being estimated on the basis of their mannose content [7,25,27].

Fourier-transform infrared (FT-IR) spectroscopy techniques have also been used for the quantification of wine polysaccharides, and a predictive model based on the quantification of mannose was proposed for MPs determination [5,28,29].

A different approach recently used by several authors [20,30] includes the use of high-resolution size-exclusion chromatography with refractive index detector (HRSEC-RID) for the separation of polysaccharides based on their molecular weight (MW). HRSEC-RID results have been shown to correlate well with GC-MS data for the total polysaccharides quantification, but given that in musts and wines high molecular weight MPs are found alongside lower molecular weight MPs, the use of size exclusion chromatography techniques for quantification of individual polysaccharide fractions resulted inaccurate [27].

Following a different approach, Quiros and colleagues [31,32] proposed a 4-steps method including the pre-fractionation of polysaccharides by size exclusion chromatography, followed by acid hydrolysis of polysaccharides, elimination of acid by weak anionic exchange solid phase extraction, and analysis of monosaccharides by ion exclusion high performance liquid chromatography (HPLC) with a refractometer as detector.

Therefore, despite numerous authors have successfully used these approaches for research purposes [5,21,28,33,34,35,36,37,38], the proposed techniques are generally long and complex and thus not suitable for MPs quantification to be used in routine wine analyses.

Lectins, carbohydrate-binding proteins with known specificity, are powerful tools for carbohydrate studies [39]. Concanavalin A, a lectin that specifically binds to mannose and glucose residues, exhibits 10 times less specificity for glucose than for mannose [39] and therefore it has been extensively used for the study of mannose-rich glycans [1,5,7,24].

An interesting method based on the biotin/avidin system has been proposed, in which lectins are biotinylated and detected with enzyme-conjugated avidin (e.g., alkaline phosphatase or horseradish peroxidase enzymes) [40]. This approach, named enzyme-linked lectin sorbent assay (ELLSA), have been successfully used to study several types of lectins, and a comprehensive description of the principle and the methods used has been reported by Wu and colleagues [41].

Despite being valid as research tools, the available methods reported in literature for MPs quantification are laborious, not suitable for high-throughput quantification, and rely on indirect methods to calculate the mannoprotein content of a wine. Moreover, the complexity and number of steps of the proposed methods do not guarantee the total recovery of the MPs of the original wine. In this study the development and validation of a novel method to quantify wine MPs are described. This method is based on a competitive indirect enzyme-linked lectin sorbent assay (CI-ELLSA) in microtiter plates, using the lectin concanavalin A as the immobilized binding agent for MPs and yeast invertase as the standard.

## 2. Results and Discussion

An official method from the international organisation of vine and wine (OIV) for MPs quantification in wine does not exist, while having one used by a large number of people in different countries would be beneficial to better characterize different wines. Every method proposed for MPs quantification seems to have drawbacks due to complexity, and possible loss of MPs material during the preparation and detection steps (e.g., chromatographic separations, calculations based on monosaccharide profiles potentially affected by hydrolysis conditions and derivatization, etc.) [5,21,28,33,34,35,36,37,38].

In contrast, the here-proposed CI-ELLSA approach use minimal sample preparation (only one precipitation step) and directly quantifies MPs without hydrolysis or derivatizations by exploiting their affinity for a specific ligand (ConA). Therefore, the results of the CI-ELLSA method were intentionally not validated against previously published methods as none of them can be considered as the reference method for MPs quantification.

In the CI-ELLSA method here developed, the fact that highly mannosylated compounds, including MPs, can be specifically bound by the lectin ConA adsorbed on microtiter plates has been exploited. To quantify these compounds a competitive assay format has been chosen, using a competitor protein with enzymatic activity, peroxidase, which is also a mannosylated enzyme [42] and therefore presents affinity for ConA. After the competitive binding to this lectin, the activity of peroxidase can be determined spectrophotometrically at 450 nm using a specific substrate, thus allowing to determine the quantity of peroxidase bound by the ConA. Due to the competition with the mannose-containing compounds present in wine, this quantity is inversely proportional to the concentration of MPs, resulting in the possibility to indirectly calculate the quantity of these compounds in unknown samples by using a four parameters regression curve, which is the most suitable systems for indirect competitive assays [43].

The final CI-ELLSA protocol described has been developed and validated by: (i) testing the response of the method to different proteins (bovine serum albumin (BSA), fetuin from fetal calf serum, and yeast invertase); (ii) testing the optimal range for the standard curve in different matrices (in binding buffer and ultra-filtered wine); (iii) validating the test on white wine samples naturally containing, or artificially spiked with, different mannoproteins/yeast invertase concentration.

### 2.1. Determination of Optimal Conditions for the Competitive Indirect Enzyme-Linked Lectin Sorbent Assay (Method Development)

Several preliminary trials have been performed in order to develop the CI-ELLSA method. Initially, the focus was on the determination of the most appropriate glycosylated compound to act as standard for the assay’s calibration curve. Given that pure MPs as those found in wine are not available commercially, yeast invertase was selected as the model for wine MPs. This glycosylated enzyme contains about 50% mannose [44] and therefore it was considered as the most suitable standard for the quantification of wine MPs.

It must be noted that yeast invertase is present in wine in combination to grape invertase that can represent 10−20% of the wine proteins [45,46]. Given that grape invertase has recently been demonstrated to contain mannose residues in almost all of the glycans potentially attached to its N-glycosylation sites [47], the here described MPs quantification system detects also this protein in addition to the cell wall MPs. However, this should be considered as an advantage of the method if the quantification of the mannose-containing species is intended for technological aims. In fact, yeast invertase and its fragments have been shown to have an haze-protective role in white wines [14,48], and to modify the foam behavior in sparkling wines [23,45,49,50].

In addition, BSA and fetuin were tested because BSA does not contain mannose, and therefore can be a suitable as the negative control, whereas the mannose content of fetuin (8–10% [51]) is about 5-fold lower than that of yeast invertase.

The plates coated with ConA were loaded with serial dilutions of the three proteins at concentrations ranging between 25 mg/L (2.5 µg/100 µL) and 0.78 mg/L (0.078 µg/100 µL).

**Figure 1:**
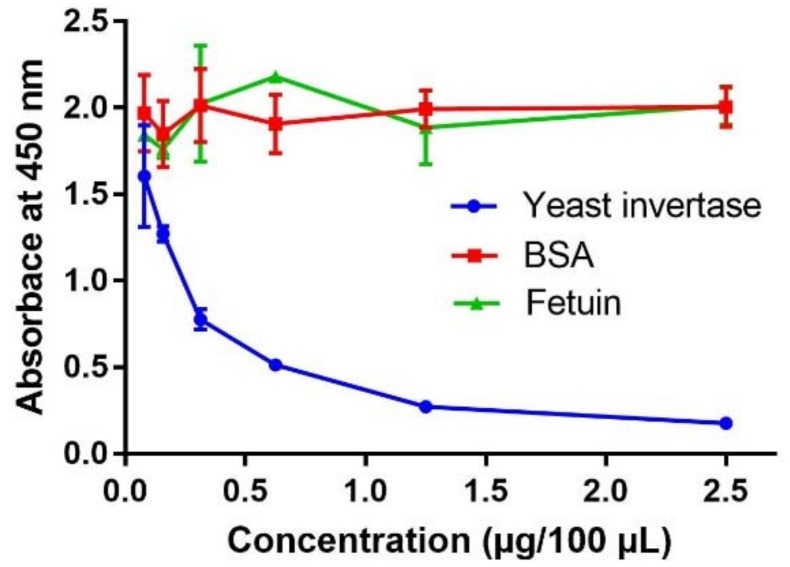
Variation in absorbance at 450 nm of for increasing concentrations of yeast invertase, fetuin and bovine serum albumin (BSA).

Results showed that both BSA and fetuin did not compete with the peroxidase, as visible by the lack of variation in absorbance at 450nm (Abs450) at increasing concentration of protein (Figure 1). This indicates that no aspecific binding to the ConA occurred and that the compounds with low mannose content, as fetuin, do not interfere with the analytical signal. Conversely, Abs450 decreased at increasing concentration of yeast invertase, indicating that this mannose-rich glycoprotein was able to compete with peroxidase for binding to the ConA adsorbed on the plates. Therefore, yeast invertase was selected as the glycoprotein for the preparation of the standard curve to be used for MPs quantification.

In order to assess whether a wine sample could be directly analyzed by CI-ELLSA, a trial was performed to investigate if the wine matrix could interfere with the final quantification of the mannose-rich compounds. To this aim, a white wine was ultra-filtered at 3 kDa molecular weight cut-off (MWCO) (ultra-filtered, UF, wine) to remove all its macromolecules, added with known amounts of yeast invertase, serially diluted in binding buffer and analyzed. In this case the results were erratic (not shown), indicating that the presence in wine of low MW compounds (<3 kDa) interfere with the ConA-yeast invertase binding. A likely candidate for this effect is ethanol, given that it has been shown to alter the lectin-carbohydrate binding affinity [52].

**Figure 2:**
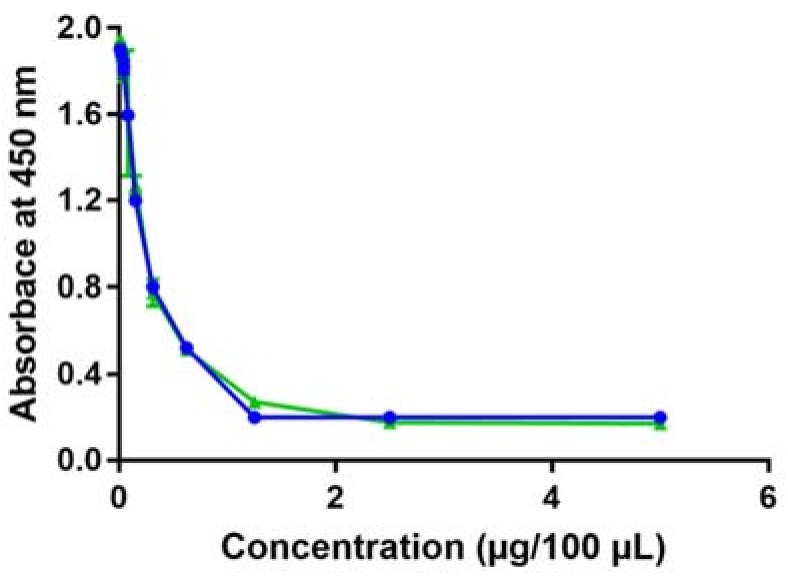
Variation in absorbance at 450 nm of serial dilutions of: ultra-filtered (UF) white wine spiked with increasing amounts of yeast invertase, precipitated with ethanol and dissolved in binding buffer (blue line); yeast invertase dissolved directly in binding buffer (green line).

In contrast, when the yeast invertase added to the UF wine sample was precipitated by cold ethanol and dissolved in binding buffer, the results were consistent with the dilution level applied (Figure 2, blue line), behaving in the same way as when yeast invertase was dissolved directly in binding buffer (Figure 2, green line). These results indicated that the wine matrix can interfere with the assay, but this interference is avoided by separating the high MW compounds from the wine matrix prior to the analysis.

### 2.2. Recovery Test in Ultra-Filtered White Wine Spiked with Yeast Invertase

The accuracy and repeatability of the proposed method were assessed in a spiking trial. In detail, the UF wine was spiked with yeast invertase at concentrations ranging from 0–12.5 mg/L. The spiked samples were added with ethanol and the formed pellet was recovered via centrifugation prior to being dissolved in binding buffer and loaded on the plate. Each spiking was replicated at least three times within each plate. The experiment was replicated six times over a period of five weeks to assess the repeatability of the method, always using the same UF wine. The obtained results (Table 1) allowed to determine the recovery by comparing the quantity of yeast invertase spiked in the samples with that found by CI-ELLSA.

In particular, the average yeast invertase recoveries for the four addition rates ranged between 84.5% for the highest addition (12.5 mg/L) and 132.8% for the lowest addition (1.5625 mg/L).

Given the nature of the method and the fact that the concentrations of the analytes are calculated using a 4-parameters logarithmic curve, for the calculation to be accurate the percentage of inhibition (*B/B*_0_) needs to lay on the linear section of the standard curve with the best results to be expected with percentages of inhibition close to *B/B*_0_ of 50% [43]. To achieve this, a serial dilution of the sample to be analyzed needs to be prepared, so that, when calculating the amount of analyte, the value of inhibition closest to a *B/B*_0_ of 50% can be selected. The calculation of MPs quantities was carried out considering the average absorbance for each dilution point so that the *B/B*_0_ values could be obtained. Figure S1 shows a representative example of the curves obtained for a yeast invertase standard and a wine sample. Samples with *B/B*_0_ outside the linear part of the curve (20–80% range of inhibition) cannot be considered reliable as the more a value is close to the tails of the sigmoidal curve the higher will be the calculation error given that this curve is logarithmic [43]. This is consistent with the data obtained for the samples showing the highest and the lowest percentages of recovery, which were the two with *B/B*_0_ towards the tails of the curve that over-estimated (e.g., 12.5 mg/L yeast invertase) or under-estimated (1.5625 mg/L yeast invertase) the real concentration of the analyte (Table 1). Therefore, for the calculation to be accurate, yeast invertase content needs to be within a range of concentration that gives *B/B*_0_ near the 50% of inhibition. In these experimental conditions the method was accurate in calculating MPs contents ranging between 3–6 mg/L of yeast invertase equivalents.

The data of Table 1 have also been used to calculate the intra-assay coefficient of variance (CV) to highlight how variable findings are. The CVs for three out of four samples are acceptable and indicated that, despite including several manual steps that could introduce small errors, the method can be used successfully for the calculation of mannosylated compounds in wine. Generally speaking, this experiment demonstrated that both the sample preparation and quantification are accurate and repeatable.

### 2.3. Mannoprotein Quantification in Different White Wine Samples

The optimized and validated method was tested on two sets of white wine samples. The first set was composed of four bottle-fermented sparkling wines produced from the same base wine and re-fermented with four different yeast strains (Table 2). Since different yeast strains can release different amounts of MPs in the wine [53], the observed differences in MPs can only be attributed to the ability of different yeast strains in releasing MPs during alcoholic fermentation and autolysis.

The MPs content of wines produced by the different yeast strain show significant differences, with values in agreement with those found in the literature by other methods [31], and ranging between 123.8 mg/L to 303.2 mg/L of MPs. The method developed was able to clearly discriminate wine samples based on their MPs content, as seen by the statistically significant difference on the measured MPs contents. MPs contents are high compared to still wines (see Table 3), a finding consistent with the method of production used that included a long period of ageing on yeast lees of 24 months and thus an enrichment of the wines in yeast autolytic products including MPs [9,10,18].

A second set of samples including white wines made from different varieties and styles was analyzed by CI-ELLSA in order to assess the suitability of the method for the quantification of MPs in wines elaborated in different ways and likely to have a broad range of MPs concentration. Wine samples were chosen based on their differences in terms of time spent in contact with yeast lees, one of the major factors contributing to the release of MPs in wines [10].

Results showed that wines differing on type, method of production and styles presented different MPs contents. In particular, the method was able to quantify MPs content as low as 18.1 mg/L for the “passito” wine to the 88.8 mg/L for the sparkling Moscato wine. However, to find relationships between wines and MPs content a wider range of wines should be considered for future investigations.

One consideration that needs to be done for the applicability of the method regards the serial dilutions that are required in order to analyze a wine. Obviously, when analyzing a wine with very high MPs content (e.g., the AWRI 1616 seen in Table 2) or very low (e.g., the Recioto Passito seen in Table 3) the dilution factor in which a *B/B*_0_ of 50% will be obtained is very different. Therefore, prior knowledge on the method of production and winemaking practices of a wine can be helpful for selecting the appropriate dilution range yielding the ideal MPs content ranging between 3–6 mg/L as seen in Table 1. However, given that wines can contain several classes of MPs having different sizes and protein/mannose ratios [1], the here proposed method does not allow to distinguish them in quantitative terms. Therefore, future trials should be done to elucidate whether the size and distribution of MPs classes in wine could result in different responses by the CI-ELLSA method.

As previously mentioned, ConA binds mannose and, at much lower extent, glucose [39]. This poses the question on whether other high MW polymers co-precipitating with the MPs could interfere with their quantification thus causing an underestimation of the MPs content of a wine. From a survey of the literature this risk seems to be extremely limited. For example, it has been shown that non-pectin polymers such as xyloglucan and xylans do not appear to be present to any substantial degree in must and wine [54]. The only exception is wines produced from botrytised grapes in which *Botrytis cinerea*-derived glucans can be present in high levels [55]. However, despite this was not the case for the wines analyzed in this study, this aspect should be taken into account for the analysis of botrytised wines. Other glucose-containing grape polysaccharides are poorly soluble in wines, and are generally degraded by microbial enzymes during fermentation [55]. Enzymatic degradation occurring during yeast autolysis is also responsible for the hydrolysis of glucans from the yeast cell wall [6], so that even for this class of compounds the risk of interfering with the method can be considered minimal. All of the aforementioned reasons, alongside with the lower affinity of ConA with glucose with respect to mannose, indicate that this risk is of interferences of other wine compounds with the quantification method is very small.

## 3. Materials and Methods

### 3.1. Materials and Sample Preparation

All reagents were analytical grade and were purchased from Sigma (Milan, Italy) unless otherwise stated. All wines to be analysed were added with four volumes of cold ethanol before being placed at −20 °C for 3 h to favour macromolecule precipitation. Subsequently, samples were centrifuged (10,000× *g*, 30 min, 4 °C), the supernatant discarded, and the obtained pellets dried under a stream of nitrogen before being dissolved in binding buffer. The volumes of wine to be precipitated and of binding buffer to dissolve the pellet were adjusted according to the expected MPs concentration.

The first set of wines analysed was made of four bottle-fermented sparkling white wines kindly donated by Plumpton Estate Winery (Plumpton, UK). These wines were all prepared starting from the same base wine blend (41% Chardonnay, 41% Pinot Meunier, 18% Pinot Noir, Vintage 2014) and bottle fermented using four different yeast strains at tirage: IOC 18-2007 (Institut Oenologique de Champagne, Epernay, France), AWRI1616, AWRI1502, and AWRI1571 (AB Mauri Ltd., North Ryde, Australia). Bottles were stored horizontally at 12–14 °C for 24 months before being riddled, disgorged and analysed. The second set of wines analysed consisted of six commercial white wines purchased in local stores and representatives of different styles and origin.

For the spiking trial a commercial white wine sample (cv. Garganega, Veneto region, Italy, Vintage 2016) was ultrafiltered at 3 kDa MW cut-off with Ultra 15 mL Centrifugal Filters (Amicon, Millipore, Billerica, MA, USA) to remove all of the macromolecules, including MPs.

### 3.2. Competitive Indirect Enzyme-Linked Lectin Sorbent Assay (CI-ELLSA)

#### 3.2.1. Preparation of Solutions

The solutions used during the CI-ELLSA assay were prepared as follows. The binding buffer consisted of 20 mM Tris-HCl buffer pH 7.4 with 0.5 M NaCl, 1 mM CaCl_2_, 1 mM MgCl_2_ and 1 mM MnCl_2_. Concanavalin A (ConA) extracted from the jack-bean *Canavalia ensiformis* was used to prepare the ConA stock solution at a concentration of 0.2 mg/mL in binding buffer. The solution used to saturate the microtiter wells was prepared by dissolving BSA at 0.2 mg/mL in binding buffer. The first washing solution was phosphate-buffered saline (PBS, 2 mM Tris-HCl, 20 mM NaCl and 0.2 mM KCl, pH 7.4). The second washing solution PBS-Tween (PBS-T) was prepared as the previous one, with the addition of 0.2% Tween (*v*/*v*). The competitive solution was prepared by dissolving horseradish peroxidase (type II, 150–250 units/mg, Sigma) at a concentration of 0.1 mg/mL in binding buffer. The substrate for the peroxidase was prepared by dissolving a table set of SigmaFast OPD (*o*-phenylenediamine dihydrochloride) in 20 mL of ultrapure water.

The stock solutions for the standard to be used for quantification was made by dissolving 50 mg/L of invertase from baker’s yeast (*S. cerevisiae* invertase, grade VII, >300 units/mg, Sigma) in binding buffer.

#### 3.2.2. Protocol of Analysis

**Figure 3:**
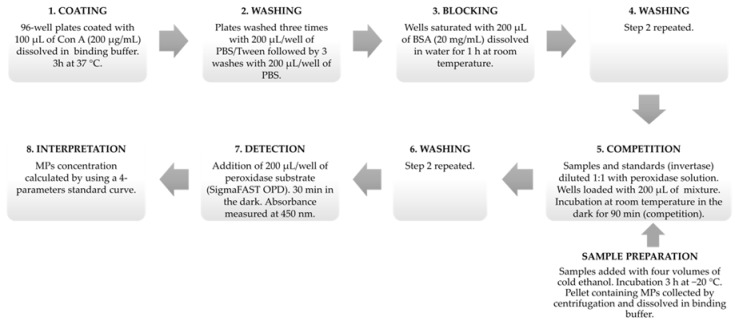
Schematic representation of the protocol of analysis of mannoproteins (MPs) by competitive indirect enzyme-linked lectin sorbent assay (CI-ELLSA).

The assay was performed by using flat-bottomed 96-well MaxiSorp plates (Nunc-Immuno, ThermoFisher, Nunc, Roskilde, Denmark). Initially, plates were coated by adding to each well 100 μL of the ConA stock solution (20 µg of ConA/well). The plates were sealed with plastic wrap and placed for 3 h at 37 °C. Afterwards plates were washed for three times with 200 μL/well of the PBS/T washing solution, followed by three washes with 200 μL/well of the PBS solution (washing step 1). After every PBS/T and PBS additions the plates were shaken for 5 s. After removing the PBS solution by turning the plates upside down and beating them onto a layer of filter paper, each well was saturated by adding 200 μL of BSA stock solution. After 1 h at room temperature the wells were washed for three times with the PBS-T washing solution (200 μL/well) followed by three further washes with the PBS solution (200 μL/well) (washing step 2) before being emptied again. At this stage, the samples and standards (prepared as described below) were diluted in 1:1 ratio (*v*/*v*) with the peroxidase solution. Each well was then added with 200 μL of this mixture, and the microplates were placed at room temperature in the dark for 1.5 h. Next, a further washing step with PBS-T and PBS was performed (washing step 3) as described above. Each well was then added with 200 μL of peroxidase substrate and incubated at room temperature in the dark. After 30 min of incubation the absorbance was measured at 450 nm by a multiscan microplate reader (Tecan Sunrise, Reading, UK), and the concentration of MPs in the unknown samples calculated by using a four-parameters standard curve prepared with known amounts of yeast invertase. A summary of the CI-ELLSA protocol of analysis is shown in Figure 3.

#### 3.2.3. Four-Parameter Standard Curve

In competitive indirect assays the concentration of the unknown analyte needs to be determined by using a 4-parameters regression curve having a sigmoidal shape [43]. Standard curves were obtained by preparing ten serial dilutions of yeast invertase in binding buffer (range 50–0.391 mg/L) and by further diluting them 1:1 (*v*/*v*) with the peroxidase solution (final range 25–0.195 mg/L).

The calculation of the concentration of the ConA-binding glycans cannot be performed directly using the absorbance readings, but need an additional step in which the percentage of inhibition (defined as *B*/*B*_0_) needs to be calculated as follows:

*B/B*_0_ = [(Abs sample − Abs min)/(Abs max − Abs min)] × 100

where *B* is calculated from the difference between the absorbance of the unknown sample and the absorbance given by the sample containing only yeast invertase (negative control) and *B*_0_ is calculated from the difference between the absorbance of the sample containing solely peroxidases (positive control) and the sample containing only yeast invertase.

To calculate the MPs content of a wine, the ethanol-precipitated polysaccharide fraction of wine samples was dissolved and serially diluted 8 times in binding buffer, and each sample loaded in the 96-well plate in triplicate. The internal standard was included in every plate by loading 4 wells with known concentrations of yeast invertase (12.5, 6.25, 3.125, 1.5625 mg/L, respectively). This allowed having a reference for the quantification of unknown samples, whose MPs content was then expressed in yeast invertase equivalents. The calculation of the four-parameters standard curve and the quantification of MPs were performed using Magellan Standard software V 4.00 (Tecan Sunrise, Reading, UK).

### 3.3. Statistical Analysis

Statistical analysis was performed using GraphPad Prism (version 7.01 for Windows, GraphPad Software, La Jolla, CA, USA), with statistical significance determined using an alpha value of 0.05.

## 4. Conclusions

It is well known that certain wine styles as bottle-fermented sparkling wines or barrel fermented white wines heavily rely on yeast autolysis and the consequent increase in MPs content for wine quality. Despite MPs being widely recognized as having a key technological and organoleptic role in wine quality, the lack of a quantification method accessible to winemakers means that a specific feedback on the results of the winemaking practices aimed at increasing or limiting the passage of MPs to the wines is not available. Knowing the MPs content of a wine will be beneficial to winemakers wanting to understand the results of their practices on final MPs concentration, so that their winemaking decisions would be better supported and guided.

The novel method proposed here shows the potential to fulfil this gap, and to become a viable tool for MPs quantification in white wines. This is supported by the data showing that MPs could be quantified in representative styles of white wines.

A key feature of CI-ELLSA is that it is specific for mannose and therefore for MPs in wine. Future work will look at setting up a high-throughput version of this method by using the support of a robot for the loading of the plates. Additionally, the method is currently being developed for application in red wines as well as for other bottle-fermented alcoholic beverages containing MPs such as beer and cider.

## Figures and Tables

**Table 1:** Recovery of yeast invertase-spiked UF wine samples (*n* = 6) ^a^.

Spiked Yeast Invertase (mg/L)	Measured Yeast Invertase (mg/L) (Mean ± SD ^b^)	Average *B*/*B*_0_^c^	Average Recovery (%)	CV ^d^ (%)
**12.5**	10.56 ± 0.85	36.8	84.5	8.1
**6.25**	6.84 ± 0.72	49.7	109.4	10.5
**3.125**	3.23 ± 0.34	71.2	103.3	10.6
**1.5625**	2.07 ± 0.36	73.4	132.8	17.2

^a^ For each concentration, three replicates were prepared and determined by the competitive indirect enzyme-linked lectin sorbent assay (CI-ELLSA). ^b^ SD, standard deviation. ^c^ Average percentage of inhibition obtained from six separate assays. ^d^ CV, intra-assay coefficient of variance.

**Table 2:** Total mannoproteins (MPs) content (expressed in mg/L of yeast invertase equivalent) of sparkling wines bottle-fermented with four yeast strains.

Yeast Strain for 2nd Fermentation	Mannoprotein Content (mg/L) (Mean ± SD)
**IOC 18-2007**	123.8 ± 7.6 ^d^
**AWRI1616**	303.2 ± 2.4 ^a^
**AWRI1502**	199.1 ± 4.2 ^b^
**AWRI1571**	167.8 ± 14.9 ^c^
*F*(3,8)	231.3
*p* value	<0.0001

Values represent the mean averages (*n* = 3). Means followed by a different letter are significantly different (*p* ≤ 0.05) according to post-hoc Tukey test. SD, standard deviation.

**Table 3:** Total mannoprotein content of wine samples elaborated from different grape varieties, region, methods of production, yeast contact and style. Values represent the mean averages (*n* = 3).

Wine Sample	Method of Production	Variety	Wine Style	MPs Content (mg/L) (Mean ± SD)
**Manzoni bianco**	White vinification with limited batonnage	Manzoni bianco	Dry white wine	67.6 ± 4.8
**Sparkling Moscato**	Asti method	Moscato bianco	Sweet sparkling wine	88.8 ± 4.8
**Recioto passito**	Withered grapes White vinification	Garganega	Sweet wine (passito style)	18.1 ± 6.0
**Prosecco 1**	Charmat	Glera	Sparkling extra dry	56.6 ± 1.8
**Prosecco 2**	Charmat	Glera	Sparkling extra dry	49.0 ± 6.9
**Prosecco 3**	Charmat	Glera	Sparkling extra brut	52.9 ± 4.1

## Supplementary Materials

Supplementary materials are available~online
