# Supplementary material for: A Novel Method for the Quantification of White Wine Mannoproteins by a Competitive Indirect Enzyme-Linked Lectin Sorbent Assay (CI-ELLSA)
Source: Molecules. 2018 Nov 23;23(12):3070. doi: 10.3390/molecules23123070 (PMC6321203; doi:10.3390/molecules23123070)
Supplement: Supplementary file 1 [file molecules-23-03070-s001.pdf]

molecules

MDPI

Appendix A

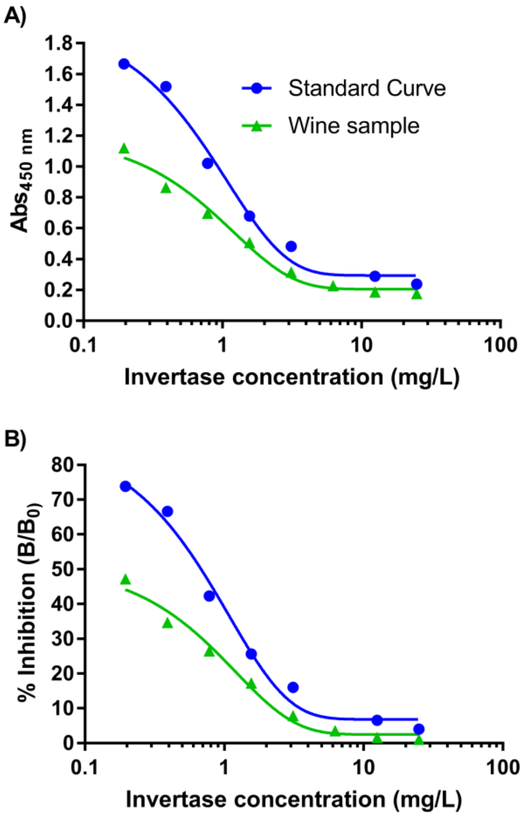

Figure S1: Calibration curve prepared with serial dilutions of yeast invertase (Standard curve, in blue) and curve prepared from a serial dilution for the determination of MPs content in a sparkling wine sample (in green). Data are expressed as Absorbance measured at 450 nm (A) and as % of inhibition expressed as B/B_0 (B).
